# Preclinical Activity of the Vascular Disrupting Agent OXi4503 against Head and Neck Cancer

**DOI:** 10.3390/cancers8010011

**Published:** 2016-01-07

**Authors:** Katelyn D. Bothwell, Margaret Folaron, Mukund Seshadri

**Affiliations:** 1 Department of Pharmacology and Therapeutics, Roswell Park Cancer Institute, Buffalo, NY 14263, USA; kbothwell@une.edu (K.D.B.); Margaret.Folaron@roswellpark.org (M.F.); 2 College of Osteopathic Medicine, University of New England, Biddeford, ME 04005, USA; 3 Department of Molecular and Cellular Biophysics and Biochemistry, Roswell Park Cancer Institute, Buffalo, NY 14263, USA; 4 Department of Oral Medicine/Head and Neck Surgery, Roswell Park Cancer Institute, Buffalo, NY 14263, USA

**Keywords:** head and neck squamous cell carcinomas, angiogenesis, vascular disrupting agents, bioluminescence imaging, magnetic resonance imaging

## Abstract

Vascular disrupting agents (VDAs) represent a relatively distinct class of agents that target established blood vessels in tumors. In this study, we examined the preclinical activity of the second-generation VDA OXi4503 against human head and neck squamous cell carcinoma (HNSCC). Studies were performed in subcutaneous and orthotopic FaDu-luc HNSCC xenografts established in immunodeficient mice. In the subcutaneous model, bioluminescence imaging (BLI) along with tumor growth measurements was performed to assess tumor response to therapy. In mice bearing orthotopic tumors, a dual modality imaging approach based on BLI and magnetic resonance imaging (MRI) was utilized. Correlative histologic assessment of tumors was performed to validate imaging data. Dynamic BLI revealed a marked reduction in radiance within a few hours of OXi4503 administration compared to baseline levels. However, this reduction was transient with vascular recovery observed at 24 h post treatment. A single injection of OXi4503 (40 mg/kg) resulted in a significant (*p* < 0.01) tumor growth inhibition of subcutaneous FaDu-luc xenografts. MRI revealed a significant reduction (*p* < 0.05) in volume of orthotopic tumors at 10 days post two doses of OXi4503 treatment. Corresponding histologic (H&E) sections of Oxi4503 treated tumors showed extensive areas of necrosis and hemorrhaging compared to untreated controls. To the best of our knowledge, this is the first report, on the activity of Oxi4503 against HNSCC. These results demonstrate the potential of tumor-VDAs in head and neck cancer. Further examination of the antivascular and antitumor activity of Oxi4503 against HNSCC alone and in combination with chemotherapy and radiation is warranted.

## 1. Introduction

Head and neck cancers affect nearly half a million individuals worldwide and approximately 50,000 individuals in the United States [1,2]. The standard of care for patients diagnosed with head and neck cancer involves a combination of surgical resection, chemotherapy and radiation therapy [3,4]. Despite developments in surgical techniques and radiation delivery methods, response rates of patients with advanced head and neck cancers remain modest. Hence, development of novel treatment strategies that exhibit therapeutic efficacy against these esthetically and functionally-debilitating cancers remains a high priority.

Angiogenesis is a pre-requisite for the sustained growth and progression of most solid tumors including human head and neck squamous cell carcinoma (HNSCC) [5,6]. As a result, targeting the vasculature of HNSCC for therapeutic benefit has received considerable attention in recent years [7,8,9]. Preclinical and clinical studies have examined the potential of anti-angiogenic agents such as Bevacizumab [7,8] or multi-targeted tyrosine kinase inhibitors such as sunitinib [9] with mixed results. Surprisingly, only a few studies have evaluated the activity of vascular disrupting agents (VDAs), a distinct class of chemotherapeutics that have been shown to result in destruction of established tumor vessels [10,11,12].

One of the widely studied VDAs is combretastatin-A4-phosphate (CA4P), an agent that causes depolymerization of the microtubules of endothelial cells resulting in their cytoskeletal rearrangement [13,14]. These cytoskeletal alterations ultimately lead to endothelial apoptosis and collapse of tumor vessels. OXi4503 is a second generation analogue of CA4P (cis-isomer of CA4P) that exhibits a similar mechanism of action to CA4P but has been shown to exhibit higher therapeutic efficacy in preclinical studies [15]. The antitumor efficacy of OXi4503 has been studied in multiple tumor models including breast [15], colon [16], renal [17], and leukemias [18] and is currently being evaluated in clinical trials [19]. However, the activity of the agent against HNSCC has not been previously investigated. To address this gap in knowledge, we examined the antivascular and antitumor activity of OXi4503 against HNSCC. Experimental studies were performed in subcutaneous and orthotopic human FaDu-luc HNSCC xenografts established in severe combined immunodeficient (SCID) mice. A combination of non-invasive imaging, histopathologic assessment and monitoring of tumor growth kinetics were utilized to assess the therapeutic potential of this agent against HNSCC.

## 2. Results

### 2.1. Acute Response of Subcutaneous FaDu-luc HNSCC Xenografts to OXi4503

We first examined the acute response of subcutaneous FaDu-luc HNSCC xenografts to OXi4503 using bioluminescence imaging (BLI). Approximately, fifteen days post implantation, SCID mice bearing subcutaneous FaDu-luc tumors were randomized into control or treatment arms (*n* = 4 controls; *n* = 6 treated). Animals in the treatment arm received a single dose (40 mg/kg, i.p.) while control animals received saline (0.1 mL, i.p.). Longitudinal BLI examination was performed at baseline (pre-treatment), 4 h and 24 h after OXi4503 treatment to assess early tumor response to VDA therapy.

**Figure 1:**
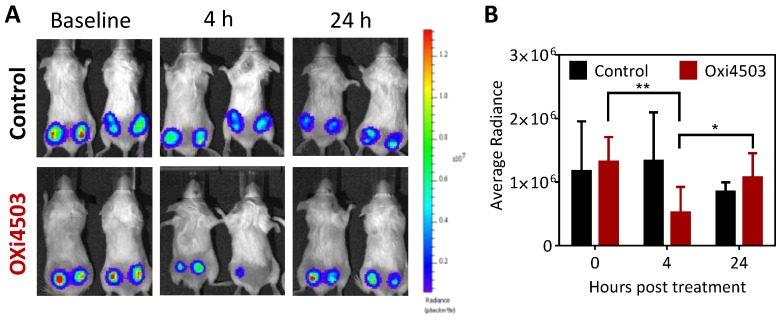
Temporal bioluminescence imaging (BLI) of FaDu-luc tumor response to OXi4503 treatment. (**A**) Panel of images represent pseudo-colorized bioluminescence images of mice in control and OXi4503 groups at baseline, 4 and 24 h post single dose vascular disrupting agent (VDA) (*n* = 4 controls; *n* = 6 treated); (**B**) Quantitative estimates of tumor radiance (mean ± standard deviation) for animals in both groups at the three time points. * denotes *p* < 0.05, ** denotes *p* < 0.01.

The panel of images shown in Figure 1A represents pseudo-colorized images of photon flux (bioluminescence signal) of control and OXi4503 treated animals at these time points. Corresponding quantitative values of radiance are shown in Figure 1B. Baseline radiance values of tumors were comparable between the control and OXi4503 arms. At 4 h post treatment, OXi4503 treated tumors exhibited a significant (*p* < 0.01) reduction in photon flux (Figure 1A,B) compared to baseline pretreatment values suggestive of VDA-induced vascular damage and tumor cell kill *in vivo*. However, this reduction in radiance of OXi4503 treated tumors was transient with values recovering by the 24 h time point (*p* < 0.05, 4 h *vs.* 24 h) to baseline levels. No significant difference in radiance values was observed in control tumors over the three time points.

### 2.2. Antitumor Activity of OXi4503 Against Subcutaneous FaDu-luc HNSCC Xenografts

**Figure 2:**
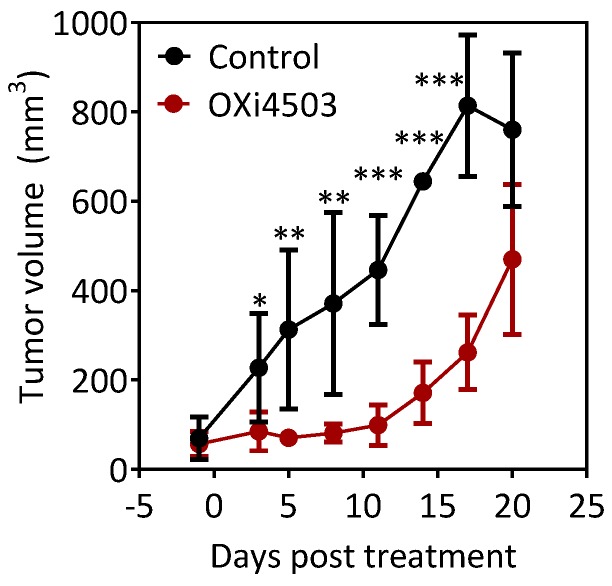
Antitumor activity of OXi4503 in the subcutaneous FaDu-luc xenograft model of human head and neck squamous cell carcinoma (HNSCC). Temporal tumor volume curves of control and OXi4503 treated mice calculated from caliper measurements. A single dose of OXi4503 (40 mg/kg, i.p.) led to a significant inhibition of tumor growth compared to untreated controls. (*n* = 4–6 per group). Values represent mean ± standard deviation at each time point. * denotes *p* < 0.05, ** denotes *p* < 0.001, *** denotes *p* < 0.0001.

Next, we examined the therapeutic efficacy of OXi4503 in the subcutaneous FaDu-luc tumor model. SCID mice bearing subcutaneous FaDu-luc tumors were assigned to control (*n* = 4) or OXi4503 arms (*n* = 6) and monitored for change in tumor growth (caliper measurements). Figure 2 shows tumor volume curves of control and OXi4503 treated mice over a three week period following treatment. As expected, tumors in untreated control animals showed a steady increase in volume over time. In comparison, treatment with a single dose of OXi4503 led to a significant inhibition of tumor growth up to 20 days of treatment.

### 2.3. Dynamic Bioluminescence Imaging (dBLI) of Orthotopic FaDu-luc Tumor Vascular Response to OXi4503

**Figure 3:**
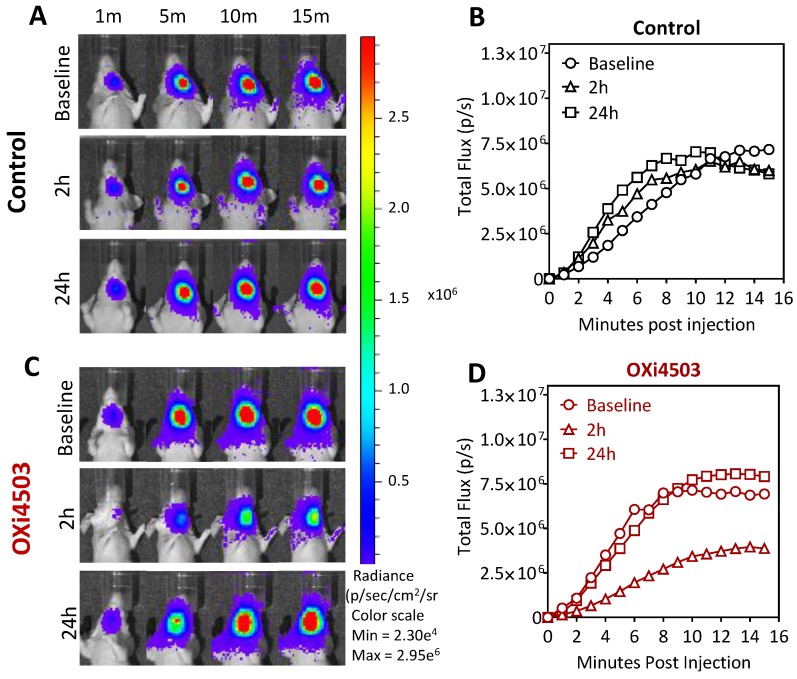
Dynamic BLI of orthotopic FaDu-luc tumor vascular response to OXi4503. Representative images of dynamic series of bioluminescence images of a saline treated control animal (**A**) and OXi4503-treated animal (**C**), at baseline, 2 h, and 24 h post treatment. A series of images were acquired up to 15 min post luciferin administration at 1 min intervals. Corresponding plots of temporal changes in flux of control (**B**) and OXi4503-treated tumors (**D**) calculated from the BLI series. Data points represent mean values from three tumors per group.

Next, we evaluated the vascular response of orthotopic FaDu-luc HNSCC xenografts to OXi4503 using dynamic BLI (dBLI). Longitudinal dBLI acquisitions were obtained at baseline, 2 h and 24 h post treatment with OXi4503 (40 mg/kg i.p.). Figure 3 shows serial bioluminescence images of a control (A) and an OXi4503-treated animal (C) bearing orthotopic FaDu-luc tumor at different times (min) post injection of the luciferin substrate. Corresponding photon flux values of the tumor in control (B) and Oxi4503 (D) treated tumors are also shown. Analysis of the dynamic series of bioluminescence images revealed a gradual increase in photon flux of control tumors over the course of 15 minutes post luciferin administration, reflective of tumor blood flow and the permeable nature of the blood vessels in the tumor (Figure 3A). Quantitative analysis of photon flux revealed the consistency in the pattern of enhancement following luciferin administration across all three time points (Figure 3B). Tumors in the VDA-treated group showed an enhancement pattern similar to control tumors at baseline (Figure 3B,D). However, at 2 h post OXi4503 treatment, a visible reduction in photon flux of orthotopic FaDu-luc tumor xenografts was observed on dBLI examination (Figure 3C). Quantitative analysis revealed a marked (~3-fold) reduction in flux at this time point (Figure 3D). However, this reduction in flux was transient with recovery to baseline levels observed at 24 h post therapy (Figure 3C,D). This transient reduction in flux followed by recovery at 24 h is indicative of acute changes in vascular integrity and blood flow to the tumor following VDA therapy.

### 2.4. Magnetic Resonance Imaging and Histopathologic Assessment of HNSCC Response to OXi4503

**Figure 4:**
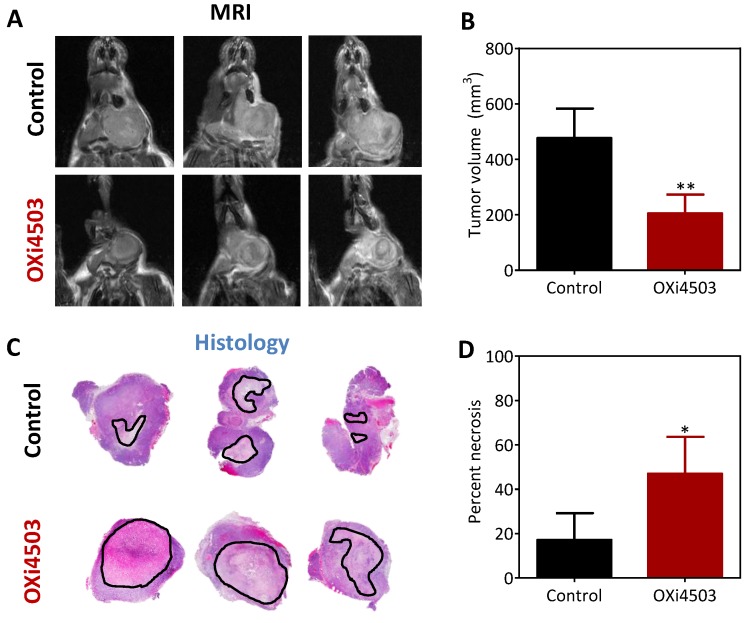
MRI and histopathologic evaluation of HNSCC response to OXi4503. (**A**) T2-weighted (T2W) MR images of control and OXi4503 treated tumors obtained at d10 post therapy. Images from three different animals (columns) in each group (rows) are shown; (**B**) Volumes of control and OXi4503 treated tumors calculated from multi-slice T2W MR images; (**C**) Corresponding photomicrographs of H&E-stained whole tumor sections of control and OXi4503 treated tumors; (**D**) Quantitative estimates of tumor necrosis calculated from whole tumor sections reported as percent necrosis (*n* = 3–4 per group). Values represent mean ± standard deviation. * *p* < 0.05; ** *p* < 0.01.

We utilized non-invasive magnetic resonance imaging (MRI) along with correlative histopathologic assessment to examine the effect of repeated dosing with Oxi4503 on HNSCC growth *in vivo*. Mice bearing orthotopic FaDu-luc tumors were administered two doses (d0, d7) of OXi4503 (40 mg/kg, i.p.) and MRI was performed on d10 (3 days post 2nd dose) to measure tumor response to therapy. The panel of images shown in Figure 4A represents non-contrast enhanced T2-weighted (T2W) images of control and OXi4503 treated animals bearing orthotopic FaDu-luc tumors. Compared to untreated controls, tumors in OXi4503 treated animals appeared smaller with evidence of hemorrhaging and necrosis. Quantitative estimates of tumor volume calculated from multi-slice T2W images confirmed a significant reduction (*p* < 0.01) in tumor growth following OXi4503 treatment compared to controls (Figure 4B). To validate our imaging data, tumors were excised from animals following completion of imaging for histopathologic (H&E) evaluation. Figure 4C shows photomicrographs of whole tumor sections of control and OXi4503 treated tumors at d10 post therapy. Histologic sections of control tumors showed microscopic foci of necrosis (*outlined in black)* associated with tumor growth (Figure 4C, *top panel*). In comparison, OXi4503 treated tumors showed extensive areas of necrosis (Figure 4C, *bottom panel*), predominantly in the central regions of the tumor. Quantitative analysis revealed a significant (*p* < 0.05) increase in tumor necrosis following OXi4503 therapy compared to controls (Figure 4D).

### 2.5. Therapeutic Efficacy of OXi4503 in the Orthotopic FaDu-luc Xenograft Model of HNSCC

**Figure 5:**
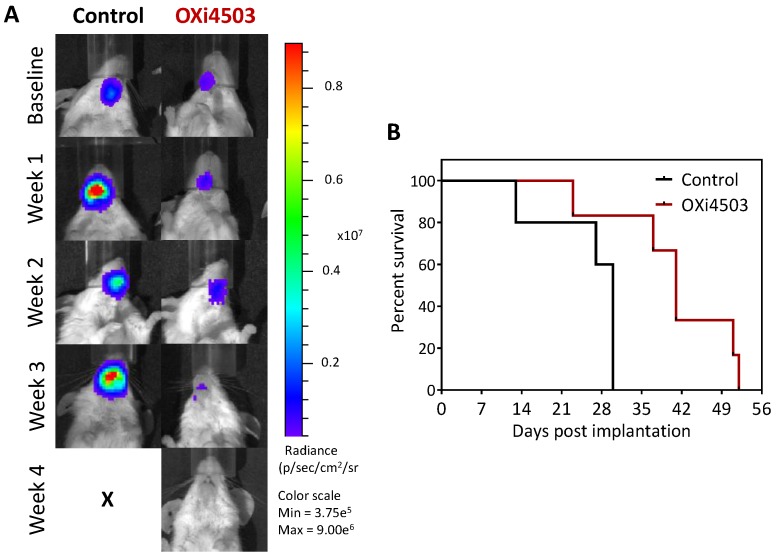
Long-term efficacy of OXi4503 against orthotopic FaDu-luc HNSCC xenografts. (**A**) Representative bioluminescence images of an untreated control and an OXi4503-treated mouse at baseline and following treatment; (**B**) Kaplan-Meier survival curves of control and OXi4503 treated animals. OXi4503 administered at a dose of 40 mg/kg, i.p. on days 6, 13 and 20 post implantation (*n* =5–6 per group).

Finally, we examined the long-term response of orthotopic FaDu-luc HNSCC xenografts to OXi4503 treatment. A separate cohort of mice bearing orthotopic FaDu-luc tumors were treated once a week for 3 weeks (40 mg/kg, i.p.) and monitored until criteria for euthanasia was met. Longitudinal BLI was performed twice a week in control and OXi4503 treated animals to monitor tumor response to therapy. Figure 5A shows longitudinal bioluminescence images of control and OXi4503 treated tumors over a 4 week period post treatment. Weekly BLI revealed a progressive increase in tumor burden in control animal. In comparison, OXi4503 treated tumors exhibited sustained inhibition of tumor growth and a visible reduction in BLI signal. Kaplan-Meier survival curves generated from time to reach threshold tumor burden (for euthanasia) demonstrated a significant survival benefit with OXi4503 treatment (*p* < 0.05) (Figure 5B). Although OXi4503 did not result in complete tumor regression in this model, VDA treatment resulted in a significant increase (*p* < 0.05) in median survival (41 days) compared to control animals (30 days).

## 3. Discussion

The overall goal of the present study was to examine the preclinical activity of the tumor VDA OXi4503, a second-generation tubulin-binding VDA against HNSCC. The working hypothesis for the study was that established vasculature of HNSCC can be safely and effectively targeted using OXi4503. Our results demonstrate a proof of concept, for the potential of OXi4503 against head and neck cancer.

In the present study, we utilized non-invasive BLI to assess the early response of subcutaneous and orthotopic FaDu-luc HNSCC xenografts to OXi4503 treatment *in vivo*. BLI is a relatively cheap and easy to use imaging technique that is widely used in preclinical research. The short image acquisition times along with the high-throughput nature of the method (*i.e*., ability to simultaneous image 3–5 animals) enables longitudinal monitoring of tumor growth in a cost-effective manner. This is an important advantage with orthotopic tumor models since these tumors are not amenable to caliper-based measurements. In addition, dynamic BLI (dBLI) methods also enable non-invasive assessment of tumor vascular function *in vivo* [20]. dBLI is based on the principle that alterations in tumor vascular integrity would affect the delivery of luciferin and as a result, affect the light emission kinetics in the tumor [20]. While the method cannot be applied clinically, given the ease of implementation, it is ideally suited for preclinical assessment of tumor response to vascular targeted therapies. Benezra *et al.*, have successfully utilized BLI to assess the response of systemic leukemia models to OXi4503 therapy in mice [18]. Studies have also validated dBLI data with dynamic contrast-enhanced MRI [21], Doppler ultrasound [22] and histopathologic measures of tumor vascularity [23]. Published studies by others [21,22,23] and us [24,25,26] and have shown that VDAs induce acute changes in tumor vascular integrity (blood flow and permeability) within a few hours of treatment. Consistent with this known mechanism of action of VDAs, we observed acute (2–4 h) changes in vascular function on dBLI following OXi4503 treatment in subcutaneous and orthotopic FaDu-luc xenografts. Although dBLI is not considered a “gold standard” for assessment of vascular function, we observed a reduction in the peak radiance value and the slope of the flux curve post OXi4503 treatment supporting our inference on vascular integrity. Nevertheless, the possibility of tumor necrosis (cell kill) post VDA treatment leading to fewer luciferase expressing tumor cells contributing to the reduced BLI signal cannot be ruled out.

Using the VDA ASA404, we have previously documented that even a single dose of VDA treatment can exhibit therapeutic efficacy against subcutaneous head and neck tumors [10]. We therefore utilized BLI to examine the response of subcutaneous FaDu-luc HNSCC xenografts to a single dose of OXi4503. The BLI results provided evidence of vascular disruption within 4 h of VDA treatment which was followed by vascular recovery at 24 h post a single dose of OXi4503. Although no significant differences in tumor volume were seen at the 24 h time point, treatment-induced vascular shutdown and tumor cell kill was sufficient to delay tumor growth compared to control animals in this model as evident from the tumor growth measurements (as early as 3–4 days post treatment). We observed significant tumor growth inhibition following treatment with a single dose of OXi4503 (40 mg/kg, i.p.). This is not surprising given that previous studies have shown that OXi4503 is more potent than the parent compound CA4P and exhibits therapeutic activity at doses ranging from 1–400 mg/kg [15,27]. Evidence from preclinical studies suggests that the tolerability, stability and solubility and mechanism of action of OXi4503 differs from the parent compound CA4P [15,27,28,29]. While the antivascular effect of OXi4503 is attributed to the destabilization and collapse of microtubules, direct tumor cytotoxicity and free radical formation have also been implicated in the antitumor efficacy of the agent [30,31]. Studies performed by us using other VDAs have demonstrated early vascular changes that occur within the 24 h period contribute to the subsequent tumor response (tumor growth inhibition, onset of necrosis) seen at 3–4 days post treatment [10,25]. The results of the present study are consistent with these previous observations.

Subcutaneous tumor models are economically feasible but are limited in their ability to reflect the influence of the microenvironment on tumor response to therapy. Expanding on our observations in the subcutaneous tumor model, we examined the vascular response of orthotopic FaDu-luc HNSCC xenografts to OXi4503. In this model system, dBLI revealed a transient reduction in luciferin delivery to tumors 2 h after OXi4503 treatment with recovery seen by 24 h. In a previous study, we have demonstrated that durable responses to VDA therapy in orthotopic models require multiple treatments [25]. We therefore examined the therapeutic impact of multiple treatments of Oxi4503 in the aggressive orthotopic FaDu-luc model. Non-invasive MRI revealed a significant reduction in tumor volume following OXi4503 after repeated dosing (7 days apart). A similar observation has been previously reported by Malcontenti-Wilson *et al.*, in which intermittent dosing of OXi4503 led to improved antitumor activity with minimal toxicity in a murine model of colorectal liver metastases [32]. In our study, histologic assessment of orthotopic FaDu-luc tumors revealed high levels of necrosis 24 h after two treatments with OXi4503 at a dose of 40 mg/kg. This is consistent with the study by Sheng *et al.*, in which tumor necrosis was observed from 6–72 h post OXi4503 treatment [29]. Importantly, in our study, treatment with OXi4503 conferred a survival advantage in the orthotopic FaDu-luc tumor model.

Taken together, our results demonstrate the potential of OXi4503 against head and neck cancer. However, the limitations of our study need to be acknowledged. Although we studied the response of subcutaneous and orthotopic xenografts to OXi4503, the observations are limited to a single HNSCC cell line (FaDu). Furthermore, given the need for immunodeficient animals, xenograft models are limited in their ability to reflect tumor-host interactions in angiogenesis and response to VDA therapy. Future studies should therefore validate these initial observations in another immunocompetent model. Finally, we examined the activity of OXi4503 as a single agent using a single dose. While the results provide important proof-of-concept, it would be important to examine the activity of OXi4503 at multiple doses in combination with standard of care regimens (chemoradiation) in multiple HNSCC models to better mimic the clinical scenario.

Several caveats to clinical translation of our findings also warrant careful consideration. First, the angiogenic dependence of most solid tumors including HNSCC is well recognized [5,6]. Yet, the role of vascular-targeted therapies in the treatment paradigm for HNSCC remains unclear. Despite heightened expectations, the clinical activity of antiangiogenic agents and VDAs in randomized clinical trials has been disappointing [33,34]. While recent failures in the Phase III setting have dampened the enthusiasm for this class of agents, several VDAs are currently in preclinical and clinical development [19]. It is worth noting that a majority of preclinical and clinical investigations on vascular targeted therapies in HNSCC have focused on antiangiogenic agents [7,8,9]. Only a few clinical trials have highlighted the potential of VDAs against head and neck cancers [35,36]. The present study adds to the limited body of literature on the potential of VDAs against HNSCC. Second, VDAs are rarely curative as single agents with most tumors developing adaptive resistance mechanisms that contribute to treatment failure. Consistent with this knowledge, our long-term response studies demonstrated tumor growth delay following OXi4503 treatment without any evidence of complete regression (cures). Hypoxia-induced upregulation of pro-angiogenic factors that leads to revascularization [37] and induction of epithelial mesenchymal transition [38] have been implicated as the survival mechanisms that contribute to tumor regrowth following VDA therapy. Shaked *et al.*, have shown that vascular damage following Oxi4503 treatment induces hypoxia which in turn upregulates pro-angiogenic factors and recruits circulating endothelial progenitor cells to the tumor resulting in a “vasculogenic rebound”. It has been shown that combining OXi4503 with chemotherapeutic agents or antiangiogenic agents can block this vasculogenic rebound and potentiate antitumor activity *in vivo* [37]*.*

Indeed, such combination approaches utilizing VDAs with chemotherapeutics, antiangiogenic agents have been investigated [25,39,40,41]. While the mechanistic rationale for dual targeting of tumor cells and the vasculature is strong, the sequence and timing of such combination treatments is a critical determinant of toxicity and efficacy [25,42]. Given the temporal nature of tumor vascular response to VDA therapy, identifying an optimal schedule and sequence becomes critical to ensure adequate drug delivery for maximal benefit [25,42,43]. Such optimization studies should be carried out in early phase clinical trials to identify the optimal dose/schedule of VDAs for further evaluation [44,45]. Clinical application of VDAs in combination with antiangiogenic agents would also require careful consideration of toxicities associated with combined use of multiple agents targeting the vasculature.

In summary, the marked enthusiasm during the early development of VDAs and antiangiogenic agents has now been transformed into skepticism and concern over the safety and efficacy of these agents. This can be at least partly attributed to disparities between preclinical studies and the patient population enrolled in clinical trials. While the results of our preliminary study are encouraging, results from preclinical studies of VDAs should be interpreted with caution. Recognizing the host related factors that affect pharmacokinetics and pharmacodynamics and tumor related factors that influence vascular phenotype, function and susceptibility to VDA therapy is critical. The future clinical utility of VDAs depends on evaluation of approaches that can safely and effectively target pathways that mediate tumor resistance to VDA therapy.

## 4. Materials and Methods

### 4.1. Tumor Model

Human head and neck squamous cell carcinoma cells transfected with the firefly luciferase reporter (FaDu-Luc) were cultured in complete Dulbecco’s modified eagle medium containing 10% fetal bovine serum and 5% penicillin-streptomycin and incubated at 5% CO_2_. Tumors were established in eight to twelve week old severe combined immunodeficiency (SCID) mice by inoculation of 1 × 10^6^ FaDu-luc cells suspended in media into the flank (subcutaneous model) or in the oral cavity (orthotopic model) as described previously [25]. Animals were housed in microisolator cags, received food and water *ad libitum* and were maintained on 12 h light/dark cycles in a HEPA-filtered environment. All experimental procedures were performed under aseptic conditions and in accordance with protocols approved by the Institutional Animal Care and Use Committee (IACUC) at Roswell Park Cancer Institute (RPCI).

### 4.2. Drug Treatment

Tumor bearing mice were treated with the vascular disrupting agent OXi4503 (Combretastatin A1 di-phosphate/CA1P). The agent was kindly provided by Dr. David Bellnier. The drug in powder form was diluted in a vehicle of 5% sodium bicarbonate, 95% phosphate-buffered saline solution at a concentration of 4 mg/mL and administered at a dose of 40 mg/kg by intraperitoneal (i.p.) injection. SCID mice bearing subcutaneous tumor xenografts were treated with a single dose of OXi4503 at 40 mg/kg i.p. while mice bearing orthotopic head and neck tumor xenografts received drug treatment once a week for 3 weeks at the same dose.

### 4.3. Bioluminescence Imaging

Bioluminescence imaging of mice was performed using the Xenogen IVIS imaging system (Caliper Life Sciences, Alameda, CA, USA) as previously described by us [25]. For BLI based monitoring of tumor burden, d-Luciferin (Gold Biotechnologies, St. Louis, MO, USA) was administered in phosphate-buffered saline and administered at a concentration of 150 mg/kg i.p. 2 min prior to imaging. For dynamic bioluminescence imaging (dBLI) assessment of vascular integrity, serial images were acquired for 15 min post luciferin (75 mg/kg, s.c.) administration at 1 minute intervals. The imaging parameters for all acquisitions were: medium binning, 1 f/stop, blocked excitation filter and an open emission filter, and a 22 cm field of view to image three mice at once. Images were processed using Living Image software (Caliper Life Sciences, Alameda, CA, USA) and reported as radiance (photon flux) or photons per second per square centimeter per square radian (p/s/cm^2^/sr).

### 4.4. Magnetic Resonance Imaging

Experimental MRI examinations were performed using a 4.7T/33-cm horizontal bore magnet (GE NMR Instruments, Fremont, CA, USA) incorporating AVANCE digital electronics (Bruker Biospec with Paravision 3.0.2; Bruker Medical Inc., Billerica, MA, USA). Animals were anesthetized using 2.5% Isoflurane (Benson Medical Industries, Markham, ON, Canada) prior to and during imaging. Coronal multi-slice, T2-weighted spin echo images incorporating RARE (rapid acquisition with relaxation enhancement) encoding were acquired to measure tumor volume as described previously described [25].

### 4.5. Histopathology

Excised tumors were fixed in 10% neutral-buffered formalin (Sigma, St. Louis, MO, USA) for hematoxylin and eosin (H&E) staining. H&E-stained slides were scanned and digitized using the Scanscope XT system (Aperio, Vista, CA, USA) and images were captured using the ImageScope software. To quantify tumor necrosis, captured fit images were uploaded into Analyze software and specified regions of interest (ROI) were created to compare the overall tumor area and the regions of necrosis, which was then reported as percent tumor necrosis [25].

### 4.6. Therapeutic Response Assessment

For animals bearing subcutaneous tumors, caliper measurements were recorded 2–3 times per week and were used to calculate tumor volume (mm^3^) using the formula, *V* = ½(L × W^2^), where L is the longest axis, and W is the axis perpendicular to L. Calculated volumes were plotted as a function of time. Prior to treatment, animals were randomized either based on tumor volume measurements or average radiance signal based on BLI. Animals were monitored and euthanized when tumor burden reached the threshold for euthanasia; subcutaneous tumors equal to or greater than 2 cm in one dimension or sustained reduction in body weight (20% or greater from baseline estimates). For the orthotopic tumor model, the criteria included sustained reduction in body weight (20% or greater from baseline measurements) or if moribund status was noted (minimal movement, labored breathing, inability to consume food or water) as per institutional protocols.

### 4.7. Statistical Considerations

All statistical analyses were performed using Graphpad Prism (GraphPad Software, San Diego, CA, USA). Values of mean ± standard deviation of the mean are reported. *p*-values < 0.05 were considered significant. Differences in tumor volumes calculated from MRI were analyzed using two-tailed unpaired *t* test. Kaplan-Meier survival curves were generated and differences in survival were analyzed using the log-rank test.

## References

[1] Patel S.G., Shah J.P. (2005). TNM staging of cancers of the head and neck: Striving for uniformity among diversity. CA Cancer J. Clin..

[2] Siegel R.L., Miller K.D., Jemal M. (2015). Cancer statistics. CA Cancer J. Clin..

[3] Bernier J., Cooper J.S. (2005). Chemoradiation after surgery for high-risk head and neck cancer patients: How strong is the evidence?. Oncologist.

[4] Cognetti D.M., Weber R.S., Lai S.Y. (2008). Head and neck cancer: An evolving treatment paradigm. Cancer.

[5] Hanahan D., Weinberg R.A. (2011). Hallmarks of Cancer: The next generation. Cell.

[6] Kyzas P.A., Cunha I.W., Loannidis J.P.A. (2005). Prognostic significance of vascular endothelial growth factor immunohistochemical expression in head and neck squamous cell carcinoma: A meta-analysis. Clin. Cancer Res..

[7] Bozec A., Sudaka A., Fischel J.L., Brunstein M.C., Etienne-Grimaldi M.C., Milano G. (2008). Combined effects of bevacizumab with erlotinib and irradiation: A preclinical study on a head and neck cancer orthotopic model. Br. J. Cancer.

[8] Argiris A., Kotsakis A., Hoang T., Worden F.P., Savvides P., Gibson M.K., Gyanchandani R., Blumenschein G.R., Chen H.X., Grandis J.R. (2013). Cetuximab and bevacizumab: preclinical data and phase II trial in recurrent or metastatic squamous cell carcinoma of the head and neck. Ann. Oncol..

[9] Machiels J., Henry S., Zanetta S., Kaminsky M.C., Michoux N., Rommel D., Schmitz S., Bompas E., Dillies A.F., Faivre S. (2010). Phase II study of sunitinib in recurrent or metastatic squamous cell carcinoma of the head and neck: GORTEC 2006–01. J. Clin. Oncol..

[10] Seshadri M., Mazurchuk R., Spernyak J.A., Bhattacharya A., Rustum Y.A., Bellnier D.A. (2006). Activity of the vascular disrupting agent 5,6-dimethylxanthenone-4-acetic acid against human head and neck carcinoma xenografts. Neoplasia.

[11] Davis P.D., Dougherty G.L., Blakey D.C., Galbraith S.M., Tozer G.M., Holder A.L., Naylor M.A., Nolan J., Stratford M.R., Chaplin D.J. (2002). ZD6126 A novel vascular-targeting agent that causes selective destruction of tumor vasculature. Cancer Res..

[12] Clemenson C., Jouannot E., Merino-Trigo A., Rubin-Carrez C., Deutsch E. (2013). The vascular disrupting agent ombrabulin (AVE8062) enhances the efficacy of standard therapies in head and neck squamous cell carcinoma xenograft models. Investig. New Drugs.

[13] Tozer G.M., Prise V.E., Wilson J., Locke R.J., Vojnovic B., Stratford M.R.L., Dennis M.F., Chaplin D.J. (1999). Combretastatin A-4 phosphate as a tumor vascular-targeting agent: Early effects in tumors and normal tissues. Cancer Res..

[14] Kanthou C., Tozer G.M. (2002). The tumor vascular targeting agent combretastatin A-4-phosphate induces reorganization of the actin cytoskeleton and early membrane blebbing in human endothelial cells. Blood.

[15] Hua J., Sheng Y., Pinney K.G., Garner C.M., Kane R.R., Prezioso J.A., Pettit G.R., Chaplin D.J., Edvardsen K. (2003). Oxi4503, a novel vascular targeting agent: effects on blood flow and antitumor activity in comparison to combretastatin A-4 phosphate. Anticancer Res..

[16] Chan L.S., Macontenti-Wilson C., Muralidharan V., Christophi C. (2007). Effect of vascular targeting agent OXi4503 on tumor cell kinetics in a mouse model of colorectal liver metastasis. Anticancer Res..

[17] Siemann D.W., Shi W. (2008). Dual targeting of tumor vasculature: combining avastin and vascular disrupting agents (CA4P or OXi4503). Anticancer Res..

[18] Benezra M., Phillips E., Tilki D., Ding B.S., Butler J., Dobrenkov K., Siim B., Chaplin D., Rafii S., Rabbany S., Bradbury M.S. (2012). Serial monitoring of human systemic and xenograft models of leukemia using a novel vascular disrupting agent. Leukemia.

[19] Clémenson C., Chargari C., Deutsch E. (2013). Combination of vascular disrupting agents and ionizing radiation. Crit. Rev. Oncol. Hematol..

[20] Sun A., Hou L., Prugpichailers T., Dunkel J., Kalani M.A., Chen X., Kalani M.Y., Tse V. (2010). Firefly luciferase-based dynamic bioluminescence imaging: A noninvasive technique to assess tumor angiogenesis. Neurosurgery.

[21] Zhao D., Richer E., Antich P.P., Mason R.P. (2008). Antivascular effects of combretastatin A4 phosphate in breast cancer xenograft assessed using dynamic bioluminescence imaging and confirmed by MRI. FASEB.

[22] Mustafa K., Alhasan L., Liu L., Lewis M.A., Magnusson J., Mason R.P. (2012). Comparison of optical and power Doppler ultrasound imaging for non-invasive evaluation of arsenic trioxide as a vascular disrupting agent in tumors. PLoS ONE.

[23] Liu L., Mason R.P., Gimi B. (2015). Dynamic and fluorescence imaging of the effects of the antivascular agent Combretastatin-A4P (CA4P) on brain tumor xenografts. Cancer Lett..

[24] Seshadri M., Spernyak J.A., Maiery P.G., Cheney R.T., Mazurchuk R., Bellnier D.A. (2007). Visualizing the acute effects of vascular-targeted therapy *in vivo* using intravital microscopy and magnetic resonance imaging: Correlation with endothelial apoptosis, cytokine induction and treatment outcome. Neoplasia.

[25] Folaron M., Kalmuk J., Lockwood J., Frangou C., Vokes J., Turowski S.G., Merzianu M., Rigual N.R., Sullivan-Nasca M., Kuriakose M.A. (2013). Vascular priming enhances chemotherapeutic efficacy against head and neck cancer. Oral Oncol..

[26] Seshadri M., Spernyak J.A., Mazurchuk R., Camacho S.H., Cheney R.T., Bellnier D.A. (2005). Tumor vascular response to photodynamic therapy and the anti-vascular agent 5,6-dimethylxanthenone-4-acetic acid: Implications for combination therapy. Clin. Cancer Res..

[27] Hill S.A., Tozer G.M., Pettit G.R., Chaplin D.J. (2002). Preclinical evaluation of the antitumor activity of the novel vascular targeting agent OXi4503. Anticancer Res..

[28] Salmon H., Siemenn D.W. (2006). Effect of the second-generation vascular disrupting agent OXi4503 on tumor vascularity. Clin. Cancer Res..

[29] Sheng Y., Hua J., Pinney K.G., Garner C.M., Kane R.R., Prezioso J.A., Chaplin D.J., Edvardsen K. (2004). Combretastatin family member OXi4503 induces vascular collapse through the induction of endothelial apoptosis. Int. J. Cancer.

[30] Madlambayan G.J., Meacham A.M., Hosaka K., Mir S., Jorgensen M., Scott E.W., Siemann D.W., Cogle C.R. (2010). Leukemia regression by vascular disruption and antiangiogenic therapy. Blood.

[31] Rice L., Pampo C., Lepler S., Rojiani A.M., Siemann D.W. (2011). Support of a free radical mechanism for enhanced antitumor efficacy of the microtubule disruptor OXi4503. Microvasc. Res.

[32] Malcontenti-Wilson C., Chan L., Nikfarjam M., Muralidharan V., Christophi C. (2008). Vascular targeting agent Oxi4503 inhibits tumor growth in a colorectal liver metastases model. J. Gastroenterol. Hepatol..

[33] Herbst R.S., Ansari R., Bustin F., Flynn P., Otterson G.A., Vlahovic G., Soh C.H., O’Connor P., Hainsworth J. (2011). Efficacy of bevacizumab plus erlotinib *versus* erlotinib alone in advanced non-small cell lung cancer after failure of standard first-line chemotherapy (BeTa): A double-blind, placebo-controlled phase 3 trial. Lancet.

[34] Lara P.N., Douillard J.Y., Nakagawa K., von Pawel J., McKeage M.J., Albert I., Losonczy G., Reck M., Heo D.S., Fan X. (2010). Randomized phase III placebo-controlled trial of carboplatin and paclitaxel with or without the vascular disrupting agent vadimezan (ASA404) in advanced non-small-cell lung cancer. J. Clin. Oncol..

[35] Sosa J.A., Elisei R., Jarzab B., Bal C.S., Koussis H., Gramza A.W., Ben-Yosef R., Gitilitz B.J., Haugen B., Karandikar S.M. (2011). A randomized phase II/III trial of a tumor vascular disrupting agent fosbretabulin tromethamine (CA4P) with carboplatin (C) and paclitaxel (P) in anaplastic thyroid cancer (ATC): Final survival analysis for the FACT trial. J. Clin. Oncol..

[36] (2000). A Phase I/II Trial of Crolibulin (EPC2407) Plus Cisplatin in Adults with Solid Tumors With a Focus on Anaplastic Thyroid Cancer (ATC). In: ClinicalTrials.gov [Internet].Bethesda (MD): National Library of Medicine (US). https://clinicaltrials.gov/ct2/show/NCT01240590.

[37] Shaked Y., Ciarrocchi A., Franco M., Lee C.R., Man S., Cheung A.M., Hicklin D.J., Chaplin D., Foster F.S., Benezra R. (2006). Therapy-induced recruitment of circulating endothelial progenitor cells to tumors. Science.

[38] Fifis T., Nguyen L., Macontenti-Wilson C., Chan L.S., Nunes Costa P.L., Daruwalla J., Nikfarjam M., Muralidharan V., Waltham M., Thompson E.W. (2013). Treatment with the vascular disruptive agent OXi4503 induces an immediate and widespread epithelial to mesenchymal transition in the surviving tumor. Cancer Med..

[39] Siemann D.W., Mercer E., Lepler S., Rojiani A.M. (2002). Vascular targeting agents enhance chemotherapeutic agent activities in solid tumor therapy. Int. J. Cancer.

[40] Horsman M.R., Siemann D.W. (2006). Pathophysiological effects of vascular targeting agents and the implications for combination with conventional therapies. Cancer Res..

[41] Del Conte G., Bahleda R., Moreno V., Damian S., Perotti A., Lassau N., Farace F., Ong M., Stimpson S.J., Tunariu N. A phase I study of ombrabulin (O) combined with bevacizumab (B) in patients with advanced solid tumors. Proceedings of the ASCO Annual Meeting.

[42] Martinelli M., Bonezzi K., Riccardi E., Kuhn E., Frapolli R., Zucchetti M., Ryan A.J., Taraboletti G., Giavazzi R. (2007). Sequence dependent antitumour efficacy of the vascular disrupting agent ZD6126 in combination with paclitaxel. Br. J. Cancer.

[43] Marysael T., Ni Y., Lerut E., de Witte P. (2011). Influence of the vascular damaging agents DMXAA and ZD6126 on hypericin distribution and accumulation in RIF-1 tumors. J. Cancer Res. Clin. Oncol..

[44] Wang E.S., Pili R., Seshadri M. (2012). Modulation of chemotherapeutic efficacy by vascular disrupting agents: Optimizing the sequence and the schedule. J. Clin. Oncol..

[45] Bahleda R., Sessa C., Del Conte G., Gianni L., Capri G., Varga A., Oprea C., Daglish B., Hospitel M., Soria J.C. (2014). Phase I clinical and pharmacokinetic study of ombrabulin (AVE8062) combined with cisplatin/docetaxel or carboplatin/paclitaxel in patients with advanced solid tumors. Investig. New Drugs.

